# Outcome Measures for Central Nervous System Evaluation in Myotonic Dystrophy Type 1 May Be Confounded by Deficits in Motor Function or Insight

**DOI:** 10.3389/fneur.2018.00780

**Published:** 2018-10-02

**Authors:** Mark J. Hamilton, John McLean, Sarah Cumming, Bob Ballantyne, Josephine McGhie, Ravi Jampana, Cheryl Longman, Jonathan J. Evans, Darren G. Monckton, Maria Elena Farrugia

**Affiliations:** ^1^West of Scotland Clinical Genetics Service, Queen Elizabeth University Hospital, Glasgow, United Kingdom; ^2^Institute of Molecular, Cell and Systems Biology, College of Medical, Veterinary and Life Sciences, University of Glasgow, Glasgow, United Kingdom; ^3^Department of Neuroradiology, Institute of Neurological Sciences, Queen Elizabeth University Hospital, Glasgow, United Kingdom; ^4^Institute of Health and Wellbeing, Gartnavel Royal Hospital, University of Glasgow, Glasgow, United Kingdom; ^5^Department of Neurology, Institute of Neurological Sciences, Queen Elizabeth University Hospital, Glasgow, United Kingdom

**Keywords:** myotonic dystrophy, neuropsychology assessment, outcome measures, small pool PCR, voxel based morphometry

## Abstract

**Background:** Central nervous system involvement in myotonic dystrophy type 1 (DM1) is associated with cognitive deficits, impaired social performance and excessive somnolence, which greatly impact quality of life. With the advent of clinical trials in DM1, there is a pressing need to identify outcome measures for quantification of central symptoms that are feasible and valid. In this context, we sought to evaluate neuropsychological and self-reported measures currently recommended by expert consensus, with particular reference to their specificity for central nervous system involvement in a moderate-sized DM1 cohort.

**Methods:** Forty-five adults with DM1 and 20 controls completed neuropsychology assessments and symptom questionnaires. Those without contraindication also underwent MRI brain, from which global gray matter volume and white matter lesion volume were quantified. CTG repeat was measured by small pool PCR, and was screened for the presence of variant repeat sequences.

**Results:** The neuropsychology test battery was well tolerated and detected impairment across various domains in the DM1 group vs. controls. Large effect sizes in the Stroop and Trail Making Tests were however attenuated by correction for basic speed, which could be influenced by dysarthria and upper limb weakness, respectively. Low mood was strongly associated with increased self-reporting of central symptoms, including cognitive impairment. Conversely, self-reported cognitive impairment did not generally predict poorer performance in neuropsychology assessments, and there was a trend toward greater self-reporting of low mood and cognitive problems in those with milder white matter change on MRI. Global gray matter volume correlated with performance in several neuropsychology assessments in a multivariate model with age and sex, while white matter lesion volume was associated with executive dysfunction reported by a proxy. Screening for variant repeats was positive in three individuals, who reported mild muscle symptoms.

**Conclusions:** Identification of outcome measures with good specificity for brain involvement in DM1 is challenging, since complex cognitive assessments may be compromised by peripheral muscle weakness and self-reported questionnaires may be influenced by mood and insight. This highlights the need for further large, longitudinal studies to identify and validate objective measures, which may include imaging biomarkers and cognitive measures not influenced by motor speed.

## Introduction

Myotonic dystrophy type 1 (DM1) is a dominantly inherited, multisystem condition (1) resulting from the abnormal expansion of a CTG trinucleotide repeat (2). The clinical phenotype is highly variable, with larger CTG repeats broadly associated with earlier onset and more severe symptoms (3). Although the phenotype presents as a clinical continuum, DM1 may be sub-categorized according to age at onset of symptoms from congenital-onset, through infantile-onset (childhood before age 10 years), juvenile-onset (childhood after 10 years), to adult- and late-onset forms (4).

Symptoms arising from central nervous system (CNS) involvement in DM1 are common (5), and vary in their impact across this clinical spectrum. In congenital-onset DM1, intellectual disability is a consistent finding, and is frequently accompanied by additional neurodevelopmental diagnoses including autism spectrum disorders (6–8). In infantile- and juvenile-onset forms, learning difficulties are often present, but are typically milder than those seen in congenital-onset DM1 (9). Educational attainment in these groups may however be further compromised by concomitant attention deficit, autism spectrum or anxiety disorders (7, 10, 11).

The CNS phenotype in adult-onset DM1 is characterized by more subtle deficits of cognition. In this group, global cognitive function is usually found to be normal when measured by standard tools such as the Mini Mental State Examination (12–14), although distribution may be shifted toward the lower end of the general population range. Case-control studies however have demonstrated impaired performance in a range of targeted neuropsychological assessments, consistent with deficits of visual perception and construction, social cognition, psychomotor speed, executive functioning, visual memory and attention, with smaller effect sizes observed in language and verbal memory domains [reviewed in (15)]. Deficits are typically present in several domains in an affected individual (16), and longitudinal studies confirm progression of cognitive symptoms with time (17–19).

Changes in personality are also described in DM1. Distinctive traits include a tendency toward apathy, and avoidant behaviors such as reluctance to seek new experiences, make new friends or form intimate relationships (20–22). Furthermore, excessive daytime somnolence is commonly reported, and may occur in the absence of sleep disordered breathing (23), suggesting a central contribution to this symptom. Imaging studies confirm structural brain changes in DM1, characterized on MRI scanning by progressive, widespread atrophy and the presence of white matter lesions (24–26). Further, diffusion tensor imaging (DTI) reveals the presence of diffuse, microstructural disruption even in apparently normal-looking white matter (27). Correlations have been described between impairment in specific cognitive domains or CTG repeat length with measures of gray matter volume (28–31), total volume of white matter lesions (VWML) (31–34) and DTI measures (29, 31–36), although findings are inconsistent between studies, and no consensus imaging biomarker for CNS involvement has yet been identified (37).

Together, cognitive difficulties, loss of drive, and excessive sleepiness conspire with physical impairments to greatly impact individuals' quality of life, affecting participation in activities related to family life, leisure, employment and self-management of healthcare (38).

Recent trials involving human participants of potential disease-modifying therapies for DM1, including antisense oligonucleotide and the GSK3β inhibitor tideglusib, emphasize clinical trial readiness as a major priority for the DM1 research community (39). With respect to CNS symptoms, the Outcome Measures in Myotonic Dystrophy (OMMYD) working group (40, 41), and DM CNS taskforce (42), have highlighted the need for a validated, consensus approach to the measurement of central symptoms in the context of clinical trials. Criteria for endorsement of outcome measures by the OMMYD group are based on the OMERACT (Outcome Measures in Rheumatology) filter, a process first devised for selection of clinical trial outcome measures for rheumatological disorders (43). The OMERACT filter requires that a measure is truthful (i.e., measures the aspect of disease that is intended), discriminates between situations of interest (for example for classification of disease states, or is sensitive to change over time), and is feasible within the constraints of the intended study.

Regarding outcome measures for CNS involvement in DM1, OMMYD has recommended four neuropsychology assessments, based on evidence from previous case control studies: the Stroop test (12, 20, 44–46), Block Design subtest from Weschler Adult Intelligence Scale-Revised (WAIS-R) (12, 44, 46, 47), FAS controlled oral word association (12, 44, 47), and Trail Making Tests A and B (12, 20, 44, 47). In addition, several self-reported symptom questionnaires have been specifically developed for use in DM1, including DM1-ActivC© (48), a measure of physical ability to complete activities of daily living, and the fatigue and daytime sleepiness scale (FDSS) (49). The Myotonic Dystrophy Health Index (MDHI) (50) allows self-rating of symptoms across a range of domains, including fatigue, cognition and social performance.

While existing data support the sensitivity of these tools to impairment in DM1, and their feasibility in the context of clinical research, no study to our knowledge has explored their specificity for brain involvement in DM1 (their “truthfulness” with respect to the OMERACT filter). We hypothesized that performance in complex neuropsychology assessments rewarding rapid completion of a manual task, such as the Trail Making test and Block Design tests, or rewarding rapid speech, such as the Stroop test, may be compromised by a more basic speed limitation that could in turn be influenced by peripheral muscle weakness in DM1. Further, we hypothesized that the impairment of insight described as part of the DM1 phenotype (51) may be particularly marked with respect to deficits of cognition and social functioning, undermining the validity of self-reporting scales for measurement of CNS disease if used in isolation.

In this context, we designed a study protocol including neuropsychology tests and self-reported symptom questionnaires recommended by OMMYD, with the aim of evaluating their suitability for use as CNS outcome measures. A correction step was applied to the Stroop, Trail Making and Block Design tests to quantify the relative contributions of basic speed limitation and higher cognitive deficits to performance in these tests. Self-reported symptoms and neuropsychology data were compared with objective measures of global CNS disease burden, measured on MRI imaging.

Correlations of outcome measure scores with CTG repeat lengths were also explored. To address the limitation of traditionally used molecular methods, that fail to take account of age-dependent somatic mosaicism of the expanded allele (52), a small-pool PCR (SP-PCR) approach was used for genotyping, that has been shown to improve correlations with age at onset of symptoms (53). In addition, since the presence of sequence interruptions (variant repeats) within the CTG repeat array, present in in around 3 to 5% of individuals with DM1, has been linked to unusually mild or atypical symptoms (54), all participants were also screened for the presence of variant repeats.

## Methods

### Recruitment and administration

Patients with DM1 attending annual review appointments at the West of Scotland Clinical Genetics Service were contacted by letter and invited to participate. Recruitment was restricted to those with adult or late onset DM1; a clear onset of DM1-specific symptoms before age 16 years or learning disability diagnosed in childhood were criteria for exclusion. Controls were recruited either from families of DM1-affected participants, or via the Scottish Health Research Register (SHARE) (55). Participants from both groups were excluded if they had a history of severe head injury, or a neurological disorder other than DM1.

Written informed consent was obtained from all participants, and the study has undergone ethical review (West of Scotland Research Ethics Committee; 15/WS/0189). DM1-affected subjects could choose between completing neuropsychology assessments and questionnaires in a hospital clinic room or in their own home. Control participants were assessed in a hospital clinic room. All neuropsychology assessments and questionnaires were administered by a single operator (MJH), following training by a Professor of Applied Neuropsychology (JJE).

### Neuropsychology tools

The neuropsychology test battery included a commercial version of the Stroop test (Golden and Freshwater© Stoelting Co. 2002), Trail Making Tests from the Delis-Kaplan Executive Function System (D-KEFS™) and the Block Design test from Weschler Abbreviated Scale of Intelligence (WASI-II). These assessments were applied and scored according to the authors' instructions. Participants also completed the Edinburgh Cognitive and Behavioral ALS Screen (ECAS) (56). ECAS was devised for use in amyotrophic lateral sclerosis, but includes cognitive domains relevant to DM1, has medium to high concurrent validity with standard tools (57) and, crucially, does not include any tasks that require manual dexterity. Finally, participants completed an FAS controlled oral word association test. Since the ECAS includes a controlled word association test for the letter “S,” two additional conditions were applied for each subject, using the letter “F,” then “A.”

### Corrections for basic speed

Participants completed three conditions of the Stroop test; a word card, a color card, and finally a color-word card. To control for basic reading speed, a predicted score for the color-word task was calculated from the raw word and color scores, using the nomogram in the test manual (58). The difference between the predicted and actual performance in color-word constituted the Interference score. In the D-KEFS Trail Making Tests, performance in the number-letter switching trail (Trail 4) was corrected by comparison to performance in Trail 5, which involved tracing a pre-defined trail marked by a dotted line. Performance in Trail 5 subtracted from Trail 4 constituted the Motor Contrast Score. In the WASI-II Block Design subtest, the authors' instructions advise graded scoring, with more points awarded for completion of designs within a shorter time. Scores calculated in line with the authors' instructions were recorded as the “standard score.” To eliminate weighting for speed, the raw number of designs correctly completed by each participant was also recorded as the “non-adjusted score.”

### Comparison of ECAS with other cognitive tests

The total score for ECAS may be sub-divided into language, verbal fluency, executive, memory and visuospatial domain subscores. Performance in four tasks contributes to the executive subdomain score; a reverse digit span, a number-letter alternation task, a social cognition test, and a sentence completion task to assess cognitive inhibition (56, 59). To further explore the validity of OMMYD-recommended tools, selected components were compared with the score or scores from ECAS intended to assess comparable cognitive domains. The Stroop color-word and interference scores were compared with the ECAS total executive subscore, and score for the cognitive inhibition task alone. The number-letter switching and motor contrast Trail Making scores were compared with the ECAS total executive subscore and number-letter alternation task alone. Block design scores were compared with the ECAS visuospatial and executive subscores. FAS oral word association score was not compared with the ECAS verbal fluency score, since both utilized the same controlled oral word association format.

### Self- and informant-reported outcomes

All participants completed Fatigue and Daytime Sleepiness Scale (FDSS) (49), Beck Depression Inventory II (BDI II) (60), visual analog scale from the Short-form McGill Pain Questionnaire (61), bodily pain items from Short Form-36 (SF-36) (62), and the Dysexecutive questionnaire from the Behavioral Assessment of the Dysexecutive Syndrome (self-DEX) (63).

DM1-affected participants additionally completed The MDHI (50), DM1-ActivC © (48), and were asked to nominate a close relative, friend or carer to complete a Dysexecutive questionnaire (informant-DEX).

### Measurement of CTG repeat expansion

Genotyping of the CTG trinucleotide repeat in DM1 participants was completed by SP-PCR as previously described (52). Four reactions, each using 300 pg blood genomic DNA template, were performed for each patient. CTG repeat lengths were estimated by comparison against DNA fragments of known length in the molecular weight marker. The lower boundary of the expanded molecules in SP-PCR was used to estimate the inherited, or “progenitor” allele length (ePAL), (52) which is the major determinant of age at onset of symptoms (53), while the region of greatest band intensity constitutes the modal allele length (MAL) at the time of sampling. Samples were also screened for presence of variant repeats within the CTG repeat array by exposure to AciI enzyme (64).

### MRI

Participants attended a single MRI session at the Glasgow Clinical Research Facility at the Queen Elizabeth University Hospital, Glasgow. Provision of transport by taxi free of charge, or reimbursements of reasonable travel expenses were offered to all participants. Height and weight were recorded prior to scanning. Imaging was undertaken using a 3T Siemens Prisma MRI scanner (Software version: VE11B. Erlangen, Germany), with a 20 channel head and neck receiver coil. Pertinent sequences for the analysis presented here were T1-w 3D MPRAGE (TR = 2,300 ms, TE = 2 ms, TI = 900 ms, flip = 10°) and T2-w SPACE dark fluid (TR = 5,000, TE = 386 ms, TI = 1800 ms, flip = 120°). The whole brain was imaged, both sequences had 1.1 mm × 1.1 mm × 1.1 mm voxels.

T1-w 3D MPRAGE and the T2-w SPACE dark fluid sequences were analyzed using a Lesion Growth Algorithm (LGA) (http://www.applied-statistics.de/lst.html) (65), from the Lesion Segmentation Toolbox (LST). The LGA creates a lesion probability map, from which the number of and total volume of white matter hyperintensities were derived.

Prior to determining major brain tissue class volumes, the T1-w 3D MPRAGE images were lesion-filled using the LST toolbox. This sought to minimize the percentage error in the tissue segmentation process due to the white matter lesions (66). Following this the filled images were then segmented (67) using SPM12 (http://www.fil.ion.ucl.ac.uk/spm/). Gray matter volume, white matter volume and cerebrospinal fluid volume were obtained. Gray matter and white matter volumes were expressed as a percentage of total intracranial volume for further analysis (GMV and WMV, respectively).

### Statistical analysis

Block Design standard score and all Stroop test subscores were converted to age-adjusted T-scores using normative data provided in the test manual. D-KEFS Trail Making scores were likewise converted to age-adjusted scaled scores using the test manual. Progenitor allele length (ePAL) was converted to a logarithm with base 10 (logPAL) for statistical analysis to achieve a normal distribution. Comparison of means was undertaken using Statistical Package for the Social Sciences (SPSS, Version 24.0; IBM 2015), Cohen's effect size was calculated using G^*^Power (version 3.1) (68) and linear regression analysis carried out using R statistics software (version 3.3.2; www.r-project.org). To address the issue of multiple comparisons, the Benjamini-Hochberg correction was applied to all linear regressions, using an on-line tool (Macdonald JH, 2014; http://www.biostathandbook.com/multiplecomparisons.html) with a false discovery rate of 0.05. All linear regression analyses and Benjamini-Hochberg correction are demonstrated in on-line Supplementary File S2.

## Results

Raw results data are provided in on-line Supplementary File S1.

### Cohort

Forty-seven individuals were recruited from the West of Scotland service. One participant was withdrawn due to an unexpected finding of a possible glial neoplasm on MRI brain. Another subject with a historical genetic diagnosis of DM1 was found to have an expanded allele of 43 repeats only on re-testing. DM1-specific features were absent on clinical evaluation, and so her diagnosis of DM1 was revised to that of a premutation carrier. This subject was excluded from the main analysis, although her data were included in linear regression analysis of imaging findings with CTG repeat length. Six subjects completed the protocol excluding MRI due to contraindications (three with permanent pacemaker, two claustrophobia and one high body mass index). Forty-five DM1-affected participants, 39 with MRI data, were therefore included in the main analysis. Twenty control subjects were also recruited; 12 from patients' families, and eight from SHARE. Comparison of clinical characteristics are summarized in Table 1. Four DM1 participants were prescribed modafinil, and none mexiletine. No participants had diabetes mellitus. In both control and DM1-affected cohorts, mean age of male, and female participants was not significantly different in independent samples *t*-test.

**Table 1:** Demographic details of DM1-affected and control cohorts.

	**DM1-affected**	**Control**	***p***
Number	45	20	–
Female: number (%)	26 (58%)	8 (40%)	0.282^†^
Age: mean (SD)	46.87 (12.37)	46.06 (13.14)	0.754^*^
Years of education: mean (SD)	14.38 (2.83)	14.48 (3.19)	0.971^*^
Smoking status Never: Former: Current (ratio)	27: 7: 11	5: 14: 1	<0.001^†^
Muscle impairment rating scale (MIRS) 1:2:3:4:5 (ratio)	3: 7: 10: 23: 2	–	–
ePAL: mean number of CTG repeats (SD)	235 (121)	–	–
MAL: mean number of CTG repeats (SD)	479 (253)	–	–

* Independent samples t-test;

† *Chi-square test*.

### Tolerability of the protocol

Stroop data were incomplete for four DM1-affected participants; one because the tool was not available, one male could not complete the color tasks due to red-green color blindness, and a third became frustrated and disengaged during the color-word task. Data from the fourth was excluded as she had a diagnosis of visual stress (Mears-Irlen syndrome), and obtained exceptionally low scores in Stroop despite above-average performance in other cognitive domains. Two DM1-affected participants declined to answer items within MDHI, as they felt these were offensive or intrusive (questions related to personality and sexual function, respectively). One DM1-affected participant declined to nominate a relative to complete the informant-DEX questionnaire, and another was unable to identify a suitable contact due to social isolation. All participants who commenced MRI scanning were able to tolerate the full imaging protocol.

### Deficits in the DM1-affected group compared with controls

Comparison of neuropsychology scores from DM1-affected participants with control participants are summarized in Table 2A. The DM1-affected group had lower scores on average in all elements of the Stroop, D-KEFS™ Trail Making, Block Design and FAS oral word association tests. The mean total score for ECAS was also lower in the DM1-affected group (*p* = 0.004), though subscores for verbal fluency and memory only approached statistical significance (*p* = 0.112, 0.085). Visuospatial and language subscores of ECAS showed a significant ceiling effect in DM1-affected participants, with 24 (53%) and 15 (33%), respectively gaining the maximum possible score in these subsections (Supplementary File S1).

**Table 2A:** Comparison between DM1-affected participants and controls in neuropsychology assessments.

	**Affected *n***.	**Control *n***.	**DM1-affected participants: Mean (SD)**	**Control participants: Mean (SD)**	**Effect size (Cohen's d)**	***P***
**STROOP TEST (T-SCORE)**						
Word task	43	20	39.42 (11.39)	48.95 (6.67)	1.021	<0.001
Color task	42	20	36.71 (10.87)	49.30 (7.23)	1.364	<0.001
Color-word task	41	20	41.95 (10.56)	53.80 (7.98)	1.266	<0.001^*^
Interference	41	20	47.34 (6.88)	51.60 (6.88)	0.619	0.022^*^
**D-KEFS**™ **TRAIL MAKING (SCALED SCORE)**						
1. Number scanning	45	20	9.00 (2.71)	11.50 (2.12)	1.028	0.001
2. Number sequencing	45	20	7.84 (3.77)	12.00 (1.59)	1.438	<0.001^*^
3. Letter sequencing	45	20	8.27 (3.86)	12.50 (2.04)	1.370	<0.001^*^
4. Number-letter sequencing	45	20	8.42 (4.25)	11.70 (2.00)	0.988	0.001^*^
5. Motor	45	20	8.96 (3.32)	12.55 (1.32)	1.421	<0.001^*^
Motor contrast score	45	20	9.51 (3.74)	9.15 (2.48)	0.113	0.221^*^
**FAS WORD ASSOCIATION**						
Number of words	45	20	37.00 (10.92)	47.10 (11.11)	0.917	0.001
**WASI-II BLOCK DESIGN**						
Standard score (T-score)	45	20	37.49 (9.88)	52.15 (8.49)	1.592	<0.001
Non-adjusted score	45	20	7.58 (2.73)	10.75 (1.41)	1.459	<0.001
**ECAS**						
Language	45	20	26.58 (1.94)	27.20 (2.07)	0.309	0.012^*^
Verbal fluency	45	20	17.51 (3.60)	18.70 (3.91)	0.317	0.112^*^
Executive	45	20	35.80 (6.17)	39.35 (5.80)	0.593	0.013^*^
Memory	45	20	17.04 (3.81)	18.90 (2.83)	0.554	0.085^*^
Visuospatial	45	20	11.13 (1.25)	11.85 (0.37)	0.781	0.010^*^
Total score	45	20	108.07 (11.74)	116.00 (9.50)	0.743	0.004^*^

Correction of performance in the Stroop color-word test for basic reading speed attenuated the Cohen's d effect size from a large 1.266 to a smaller, though still significant 0.619 compared with controls. Performance on the D-KEFS™ Trail Making number-letter switching task was no longer significantly different after controlling for basic motor speed (Motor contrast score; *p* = 0.221). In the Block Design subtest, the large effect size remained when weighting for speed was eliminated (standard score vs. non-adjusted score, Cohen's *d* = 1.592 vs. 1.459, respectively).

DM1-affected participants reported greater fatigue, lower mood and greater pain on FDSS, BDI II, and McGill visual analog scales, respectively (Table 2B). There was a trend toward greater everyday executive dysfunction as measured by the self-DEX questionnaire, and greater pain reported by SF-36, though these differences did not reach statistical significance (*p* = 0.102, 0.061). Levels of low mood, fatigue and pain that could be considered clinically significant were reported frequently in DM1-affected participants. Thirteen (29%) had a BDI II score greater than 13, 27 (60%) had an FDSS score greater than two SDs above the mean score of controls, and 16 (36%) rated bodily pain as “moderate” or greater on SF-36.

**Table 2B:** Summary of self-reported symptom scores of DM1-affected participants and controls.

	**Affected *N***	**Control *N***	**DM1-affected participants: Mean (SD)**	**Control participants: Mean (SD)**	**Effect size (Cohen's D)**	***p***	
Self-DEX	45	20	17.89 (11.60)	12.70 (8.05)	0.520	0.102^*^	↑
BDI II	45	20	11.22 (8.76)	5.05 (5.09)	0.858	0.001^*^	↑
FDSS Centile Score	45	20	37.47 (15.59)	17.00 (9.03)	1.607	<0.001^*^	↑
SF-36 Pain Items	45	20	4.76 (2.48)	3.45 (1.61)	0.627	0.061^*^	↑
McGill Pain Scale	45	20	22.18 (24.03)	9.20 (13.73)	0.663	0.046^*^	↑
DM1-ActivC centile	45	–	69.78 (20.00)	–	–	–	↓
MDHI total	45	–	27.78 (21.66)	–	–	–	↑

Comparison of means was carried out as an independent samples t-test if data were normally distributed in both groups (defined as p > 0.05 in Shapiro Wilk test of normality). If data were not normally distributed in one or both groups, a non-parametric Mann Whitney U-test was applied. P-values that relate to a non-parametric test are marked *.

*BDI II, Beck Depression Inventory II; D-KEFS™, Delis Kaplan Executive Frontal System; ECAS, Edinburgh Cognitive and Behavioral ALS screen; FDSS, Fatigue and Daytime Sleepiness Scale; MDHI, Myotonic dystrophy health index; WASI-II, Weschler Abbreviated Scale of Intelligence. Direction of the arrows indicates the trend that is associated with more severe symptoms*.

### Comparison of ECAS with other cognitive assessments

Performance in the Stroop color-word task did not significantly correlate with ECAS total executive subscore, or score in the sentence completion/cognitive inhibition task alone. The same was true for Stroop Interference score. Performance in the D-KEFS™ number-letter switching trail did not correlate with the number-letter alternation task of ECAS alone, but had a relatively weak positive correlation with total ECAS executive subscore (*p* = 0.006, Adj *R*^2^ = 0.141). The D-KEFS™ motor contrast score did not correlate with the ECAS number-letter alternation task or the total executive subscore.

Block Design standard score was positively correlated with both the ECAS visuospatial subscore (*p* = 0.008, Adj *R*^2^ = 0.134) and the ECAS executive subscore (*p* = 0.011, Adj *R*^2^ = 0.122). Similarly, the Block Design non-adjusted score correlated weakly with the ECAS visuospatial subscore (*p* = 0.024, Adj *R*^2^ = 0.092) and with the executive subscore (*p* = 0.009, Adj *R*^2^ = 0.127). Only the relationship between the Block Design standard score and ECAS visuospatial subscore remained significant after Benjamini-Hochberg correction. Of note, correlations with the ECAS visuospatial score were likely hampered by the ceiling effect previously described, in that 53% of DM1-affected subjects gained the maximum possible points for this subsection.

### Relationships between self-reported symptoms

**Figure 1:**
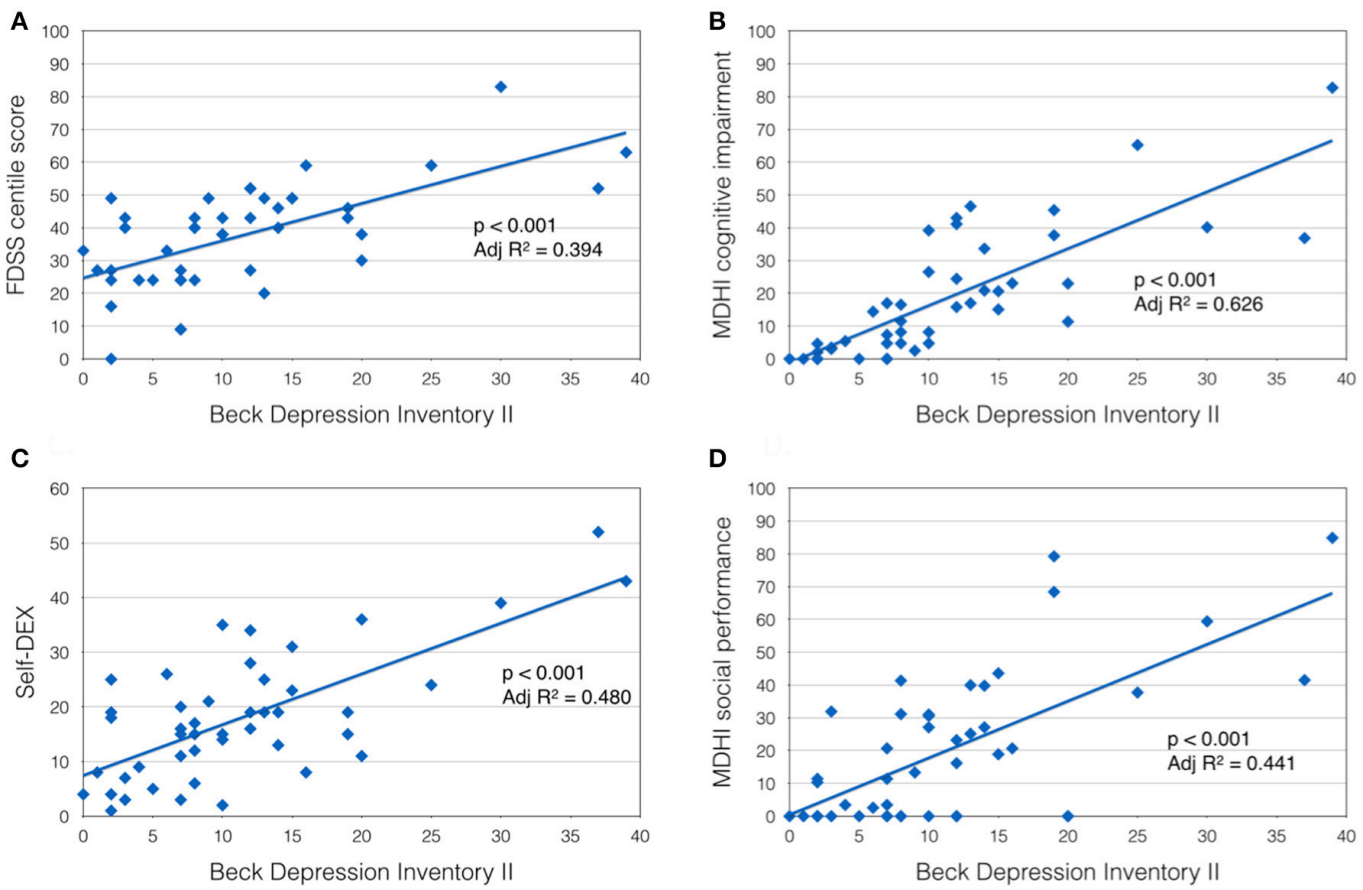
Beck Depression Inventory II score was positively correlated with **(A)** Fatigue Daytime Sleepiness Scale centile score; **(B)** MDHI cognitive impairment subscore; **(C)** self-dysexecutive (DEX) score; and **(D)** MDHI social performance subscale.

In the DM1-affected group, significant co-linearity was observed between self-reported scales of fatigue, pain and low mood. BDI II score correlated positively with FDSS score (*p* < 0.001, Adj *R*^2^ = 0.394; Figure 1A), and with McGill pain scale (*p* < 0.001, Adj *R*^2^ = 0.255). In turn, FDSS also correlated with McGill pain scale (*p* < 0.001, Adj *R*^2^ = 0.321).

A highly significant relationship was also observed between mood (BDI II score) and self-reported CNS symptoms. Patients with more symptoms of depression tended to report more cognitive problems (MHDI cognitive impairment subscale; *p* < 0.001, Adj *R*^2^ = 0.626), everyday executive difficulties (self-DEX; *p* < 0.001, Adj *R*^2^ = 0.480) and impaired social performance (MDHI social performance subscale; *p* < 0.001, Adj *R*^2^ = 0.441; Figures 1B–D). Subjects' rating of their own everyday executive dysfunction (self-DEX) correlated significantly, but weakly with the same scale completed by a proxy (informant-DEX; *p* = 0.006, Adj *R*^2^ = 0.147).

Comparison of MDHI subscores with other self-rating scales for similar themes showed good correlations. Mobility (*p* < 0.001, Adj *R*^2^ = 0.746), upper extremity function (*p* < 0.001, Adj *R*^2^ = 0.531) and ability to do activities (*p* < 0.001, Adj *R*^2^ = 0.684) subscales all correlated inversely with DM1ActivC© score as expected. BDI II score correlated with the emotional issues subscale (*p* < 0.001, Adj *R*^2^ = 0.611), FDSS centile score correlated with the fatigue subscale (*p* < 0.001, Adj *R*^2^ = 0.504), and both SF-36 pain items and McGill pain scale correlated well with MDHI pain subscale scores (*p* < 0.001, Adj *R*^2^ = 0.524, and Adj *R*^2^ = 0.582, respectively).

### Self-reported symptoms and cognitive performance

Self-DEX, BDI-II, FDSS and McGill pain scores alone did not correlate with performance in any of the neuropsychology assessments. The cognitive impairment subscore of MDHI was inversely correlated with Stroop Interference score only (*p* = 0.009, Adj *R*^2^ = 0.142).

Greater physical impairment measured by DM1ActivC© was significantly associated with poorer performance in the visual scanning (*p* = 0.001, Adj *R*^2^ = 0.198) and motor task (*p* < 0.001, Adj *R*^2^ = 0.305) of the D-KEFS™ Trail Making Tests, as well as total standard score (*p* < 0.001, Adj *R*^2^ = 0.229) and non-adjusted score (*p* < 0.001, Adj *R*^2^ = 0.210) of the Block Design subtest. Executive impairment rated by a proxy (Informant-DEX) showed an inverse correlation with Stroop word task score only (*p* = 0.003, Adj *R*^2^ = 0.181).

### Genotype-phenotype correlations

In univariate analysis, logPAL did not correlate with performance in any of the neuropsychology assessments, nor with any self-reported symptoms. Since logPAL represents an estimation of CTG repeat size at conception, its influence on phenotype would be expected to be age-dependent. Hence we also explored correlations with logPAL in a multivariate model [age + logPAL + (age^*^logPAL)], demonstrating a significant correlation with the MDHI mobility subscale (*p* = 0.008, Adj *R*^2^ = 0.194) and block design non-adjusted score (*p* = 0.005, Adj *R*^2^ = 0.212) only.

Increasing MAL significantly correlated with greater physical impairment measured by MDHI mobility subscale (*p* = 0.001, Adj *R*^2^ = 0.211) and DM1-ActivC© score (*p* = 0.006, Adj *R*^2^ = 0.143), as well as poorer performance in the Block Design standard score (*p* = 0.003, Adj *R*^2^ = 0.168).

AciI enzyme digest identified three individuals as carrying variant trinucleotide repeats; a 22 year old female, 33 year old male, and a 36 year old male. Their neuropsychology and imaging data were not remarkably different to other DM1-affected individuals of similar age (online Supplementary File S1). However all reported minimal physical impairment due to their DM1 symptoms (DM1ActivC© centile score 100, 88, and 93), despite an ePAL of 251, 217, and 158 repeats, respectively.

### MRI

**Figure 2:**
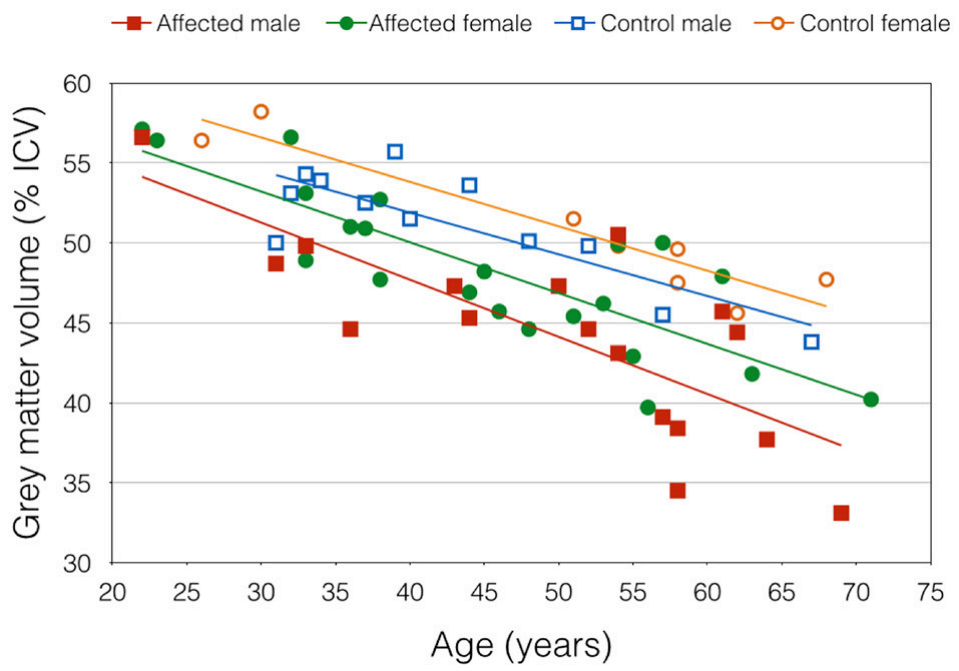
Gray matter volume, expressed as percentage of total intracranial volume, plotted against age. Trend lines demonstrate an apparent sex effect that was exaggerated in DM1-affected participants.

Mean GMV was lower in the DM1-affected participants compared with controls as expected (46.5 vs. 51.0%; *p* = 0.003). Within the DM1 cohort only, mean GMV was also significantly lower in males compared with females (44.2 vs. 48.4%; *p* = 0.023; Figure 2), despite sexes being well matched for age, and mean ePAL being significantly higher in females who underwent imaging (265 vs. 171 repeats; *p* = 0.004). A possible sex effect in the DM1 group was further evidenced by improvement of the inverse correlation between GMV and age (Adj *R*^2^ = 0.622) by inclusion of both age and sex in a multivariate model (Adj *R*^2^ = 0.665). The model improved further with inclusion of age, sex and logPAL (*n*. = 40 including subject with premutation; Adj *R*^2^ = 0.697; Table 3).

**Table 3:** Significance level of predictor variables in multiple linear regression model GMV ~ age + sex + logPAL, using data from 39 DM1-affected individuals and one premutation carrier.

**Variable**	**Standardized beta coefficient**	***p***
Age (years)	−0.815	<0.001
Sex (male = 0; female = 1)	0.322	0.002
logPAL	−0.245	0.018

Gray matter volume alone did not significantly correlate with performance in neuropsychology assessments, except the Stroop word and color tasks. Given the observed sex differences in GMV, and that our cohort was highly heterogeneous in age, we repeated linear regression analyses including sex and age as covariates, which improved correlations with several measures, although most were non-significant after correction for multiple comparisons (Table 4).

**Table 4:** Linear regression analysis between GMV and neuropsychology assessment scores in DM1-affected participants only, demonstrating improvement with multivariate models including sex and age.

	**Age** **Adj *R*^2^**	**GMV Adj *R*^2^**	**GMV + sex Adj *R*^2^**	**GMV + sex + age**
**STROOP TEST (T-SCORE)**				
Word task	ns	0.171	0.200	*p* = 0.003 Adj *R*^2^ = 0.280
Color task	ns	0.316	0.367	*p* < 0.001 Adj *R*^2^ = 0.399
Color-word task	ns	(0.101)	0.287	*p* < 0.001 Adj *R*^2^ = 0.353
Interference	ns	ns	0.275	*p* = 0.006 Adj *R*^2^ = 0.257
**D-KEFS**™ **TRAIL MAKING (SCALED SCORE)**				
1. Number scanning	ns	ns	ns	*p* = 0.013 (Adj *R*^2^ = 0.199)
2. Number sequencing	ns	ns	(0.173)	*p* = 0.007 Adj *R*^2^ = 0.232
3. Letter sequencing	ns	(0.089)	0.232	*p* = 0.002 Adj *R*^2^ = 0.276
4. Number-letter sequencing	ns	ns	(0.135)	*p* = 0.043 (Adj *R*^2^ = 0.137)
5. Motor	ns	ns	ns	ns
Motor contrast score	ns	ns	ns	ns
**FAS WORD ASSOCIATION**				
Number of words	ns	ns	ns	*p* = 0.006 Adj *R*^2^ = 0.234
**WASI-II BLOCK DESIGN**				
Standard score (T-score)	ns	ns	(0.162)	*p* = 0.041 (Adj *R*^2^ = 0.140)
Non-adjusted score	0.140	(0.105)	0.225	*p* = 0.009 (Adj *R*^2^ = 0.214)
**ECAS**				
Language	ns	ns	ns	ns
Verbal fluency	(0.097)	ns	ns	*p* = 0.006 Adj R^2^ = 0.232
Executive	ns	(0.124)	(0.126)	ns
Memory	(0.070)	ns	ns	ns
Visospatial	ns	ns	ns	ns
Total score	ns	ns	ns	ns

*ns, not significant (p > 0.05) before correction for multiple comparisons. Adj R^2^ values in brackets were no longer significant after Benjamini-Hochberg correction*.

To further explore the relative effect of GMV and muscle impairment on cognitive performance, while controlling for other factors, DM1-ActivC© score was then added to this multivariate model, to give: score ~ age + sex + GMV + DM1-ActivC© (Table 5). Addition of DM1-ActivC© improved the fit of the model, reflected by an increase in Adj *R*^2^, for the Stroop color and word tasks, Trails 1–5 of the D-KEFS Trail Making Tests and both the Block Design standard and non-adjusted scores. The contribution of DM1-ActivC© score to the model reached statistical significance (at *p* < 0.05 without correction for multiple testing) in the D-KEFS number scanning and motor trails, as well as the Block Design standard score.

**Table 5:** Standardized beta coefficients and significance of predictor variables in the model Score ~ age + sex + GMV + DM1-ActivC.

	**Age**	**Sex**	**GMV**	**DM1-ActivC©**	**Whole model Adj *R*^2^ (*p*)**
**STROOP TEST (T-SCORE)**					
Word task	*p* = 0.023 Beta = 0.521	*p* = 0.196 Beta = −0.206	*p* = 0.001 Beta = 0.862	*p* = 0.074 Beta = 0.272	0.327 (*p* = 0.002)
Color task	*p* = 0.080 Beta = 0.361	*p* = 0.100 Beta = −0.244	*p* < 0.001 Beta = 0.884	*p* = 0.123 Beta = 0.219	0.425 (*p* < 0.001)
Color-word task	*p* = 0.048 Beta = 0.461	*p* = 0.002 Beta = −0.514	*p* = 0.001 Beta = 0.868	*p* = 0.903 Beta = 0.018	0.333 (*p* = 0.002)
Interference	*p* = 0.650 Beta = 0.110	*p* = 0.002 Beta = −0.566	*p* = 0.050 Beta = 0.517	*p* = 0.668 Beta = −0.069	0.238 (*p* = 0.014)
**D-KEFS**™ **TRAIL MAKING (SCALED SCORE)**					
1. Number scanning	*p* = 0.001 Beta = 0.747	*p* = 0.849 Beta = −0.028	*p* = 0.001 Beta = 0.775	*p* = 0.001 Beta = 0.506	0.410 (*p* < 0.001)
2. Number sequencing	*p* = 0.040 Beta = 0.497	*p* = 0.101 Beta = −0.434	*p* = 0.013 Beta = 0.653	*p* = 0.089 Beta = 0.267	0.274 (*p* = 0.005)
3. Letter sequencing	*p* = 0.061 Beta = 0.444	*p* = 0.015 Beta = −0.405	*p* = 0.003 Beta = 0.782	*p* = 0.147 Beta = 0.223	0.300 (*p* = 0.003)
4. Number-letter sequencing	*p* = 0.269 Beta = 0.286	*p* = 0.109 Beta = −0.288	*p* = 0.032 Beta = 0.609	*p* = 0.364 Beta = 0.154	0.134 (*p* = 0.064)
5. Motor	*p* = 0.309 Beta = 0.237	*p* = 0.731 Beta = −0.055	*p* = 0.461 Beta = 0.182	*p* < 0.001 Beta = 0.603	0.299 (*p* = 0.003)
Motor contrast score	*p* = 0.714 Beta = 0.097	*p* = 0.156 Beta = −0.261	*p* = 0.074 Beta = 0.514	*p* = 0.053 Beta = −0.343	0.088 (*p* = 0.130)
**FAS WORD ASSOCIATION**					
Number of words	*p* = 0.001 Beta = 0.917	*p* = 0.419 Beta = −0.136	*p* = 0.004 Beta = 0.805	*p* = 0.604 Beta = 0.083	0.218 (*p* = 0.014)
**WASI-II BLOCK DESIGN**					
Standard score (T-score)	*p* = 0.650 Beta = 0.111	*p* = 0.079 Beta = −0.302	*p* = 0.162 Beta = 0.370	*p* = 0.047 Beta = 0.328	0.213 (*p* = 0.015)
Non-adjusted score	*p* = 0.589 Beta = −0.127	*p* = 0.099 Beta = −0.271	*p* = 0.266 Beta = 0.280	*p* = 0.052 Beta = 0.307	0.277 (*p* = 0.004)
**ECAS**					
Language	*p* = 0.181 Beta = 0.364	*p* = 0.094 Beta = 0.316	*p* = 0.627 Beta = 0.140	*p* = 0.226 Beta = 0.215	0.048 (*p* = 0.229)
Verbal fluency	*p* = 0.002 Beta = 0.824	*p* = 0.559 Beta = 0.098	*p* = 0.032 Beta = 0.580	*p* = 0.696 Beta = −0.063	0.213 (*p* = 0.016)
Executive	*p* = 0.254 Beta = 0.259	*p* = 0.265 Beta = 0.198	*p* = 0.074 Beta = 0.500	*p* = 0.273 Beta = 0.186	0.135 (*p* = 0.062)
Memory	*p* = 0.203 Beta = −0.352	*p* = 0.270 Beta = 0.209	*p* = 0.475 Beta = −0.209	*p* = 0.300 Beta = 0.187	0.021 (*p* = 0.328)
Visos*p*atial	*p* = 0.871 Beta = 0.046	*p* = 0.521 Beta = −0.125	*p* = 0.335 Beta = 0.292	*p* = 0.883 Beta = 0.027	0.048 (*p* = 0.687)
Total score	*p* = 0.146 Beta = 0.386	*p* = 0.169 Beta = 0.250	*p* = 0.113 Beta = 0.449	*p* = 0.294 Beta = 0.181	0.104 (*p* = 0.102)

**Figure 3:**
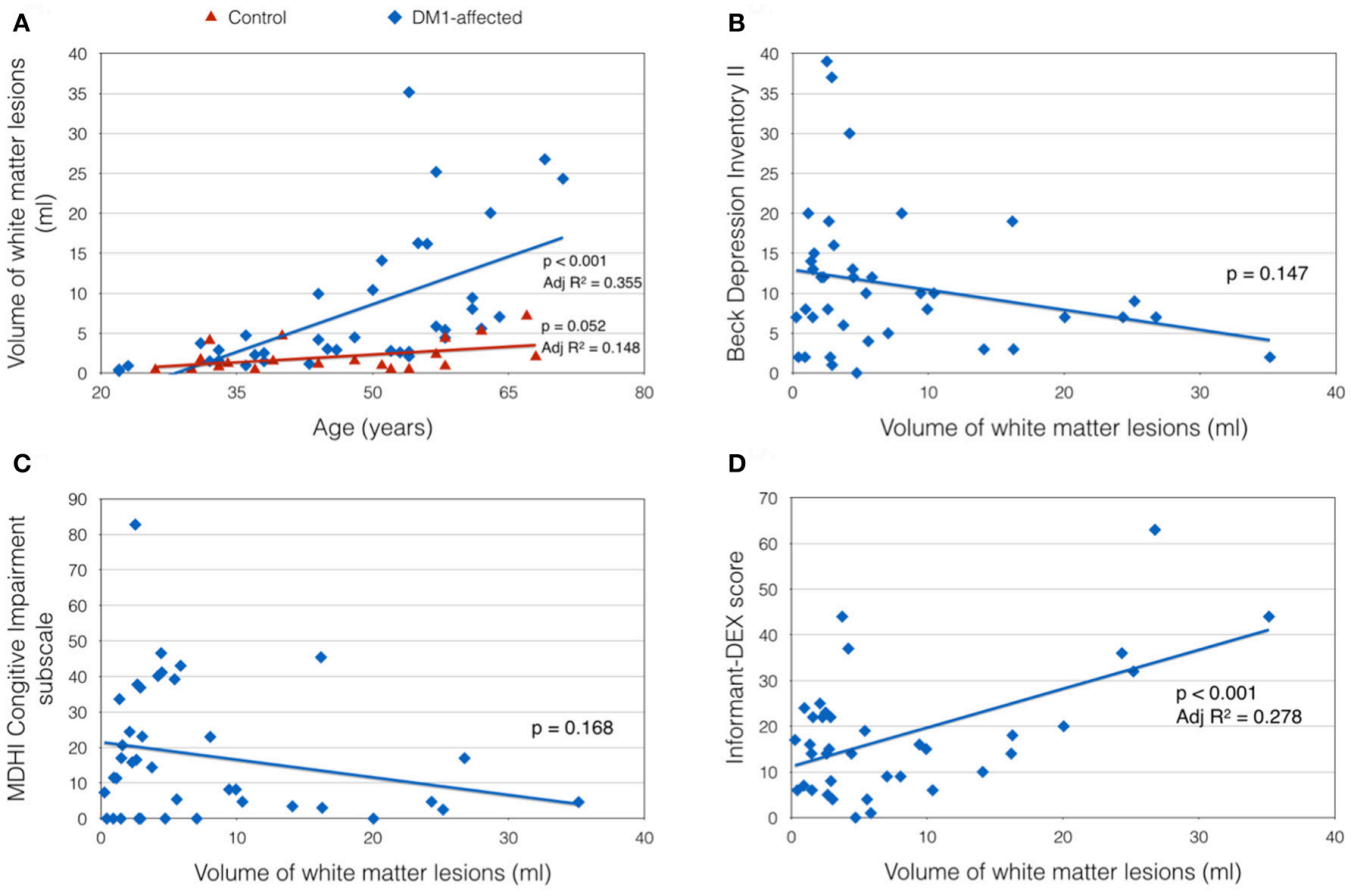
Clinical correlates of total white matter lesion volume (VWML). **(A)** VWML is increased in DM1-affected subjects compared with controls, and is age-dependent. **(B,C)** Demonstrate trends toward higher reporting of depression and cognitive impairment symptoms respectively in patients with lower VMWL. **(D)** Demonstrates a positive relationship between VWML and everyday executive dysfunction reported by a proxy (informant-DEX).

Total VWML also increased with age in DM1-affected subjects (*p* < 0.001, Adj *R*^2^ = 0.355; Figure 3A). This model did not improve with inclusion of sex, CTG repeat length (logPAL or MAL), BMI, or smoking status in a multivariate model. VWML did not correlate with performance in any of the neuropsychology assessments measured.

There was a trend toward greater depression and self-reported cognitive impairment scores in patients with lower VWML (Figures 3B,C). Higher score in the informant-DEX was significantly associated with greater VWML (*p* = 0.001, Adj *R*^2^ = 0.278) (Figure 3D).

## Discussion

In this study, a range of CNS outcome measures currently recommended for use in clinical trials were applied to a moderate-sized cohort of adults with DM1, and compared against age-matched controls. Correlations were explored with CTG repeat length and MRI evidence of global DM1-related brain changes. The results highlight several important considerations for CNS outcome measure selection in the context of DM1 clinical trials.

The feasibility of any protocol for DM1 clinical trials must be considered in the context of the physical limitations, fatigability and behavioral traits present in such cohorts (38), since these factors could result in a lower threshold for poor compliance or withdrawal compared with other groups. Despite this, the test protocol we describe was not significantly compromised by disengagement or inability to complete tasks. Furthermore, the neuropsychology test battery was apparently sensitive to impairment in DM1, detecting effect sizes of ~ −0.5 to −1.5 standard deviations compared with controls, with smaller effects in verbal and memory domains, consistent with the profile described in DM1 (16). These findings therefore support the feasibility and sensitivity of the neuropsychology assessments and self-reported questionnaires described for use in clinical studies.

Specificity of the neuropsychology battery for central vs. peripheral effects of DM1 was less clear, however. The Stroop and Trail Making Tests are commonly used in clinical and research contexts, and are broadly considered measures of higher, executive cognitive functions (69). The color-word task of the Stroop requires the subject to suppress a habitual impulse (to say the written word), and instead perform an unfamiliar task (saying the color of ink), while the number-letter switching task of the Trail Making test demands cognitive flexibility to switch repeatedly between two unrelated sequences. In reality however, both are complex tasks, and performance depends on additional domains including attention and basic processing speed (70, 71). In both tests, comparison with controls showed a similar, large effect size for a simplified version of the task (the color and word cards of the Stroop test, and motor task of the Trail Making Tests) as for the key executive component (the color-word card and number-letter switching trail). Correction of the Stroop color-word score for basic reading speed reduced this large effect size to a moderate Cohen's d value, closer to that of the executive subscore of ECAS. In the D-KEFS Trail Making Tests, correction of the number-letter switching score for basic motor speed eliminated any significant difference compared to controls, suggesting that a more basic speed limitation is the major contributor to poorer performance in the DM1-affected group.

A speed limitation affecting the simpler conditions of the Stroop or Trail Making Tests has been observed in some previous DM1 studies (12, 20, 45, 47). The nature of this limitation has not been specifically explored, though it has generally been held to reflect a more global cognitive impairment, or deficit in basic processing (72). Slowing of basic processing is a key feature of cognitive aging in the general population (73), and has been linked to reduced integrity of white matter and loss of gray matter volume in older adults (74, 75), including reduced integrity of fronto-striatal white matter tracts, and volume of subcortical structures (76). These structural changes are likely to be accelerated as part of the global brain changes seen in DM1, hence an exaggeration of the normal decline in basic speed might be predicted. The fact that we detected a basic speed limitation affecting both manual and vocal modalities could be consistent with a central, domain-general cause. A central slowing of information processing could further be speculated to underlie features of adynamia or apparent apathy that are frequently described in in DM1 (77).

On the other hand, some authors have questioned whether physical limitations due to DM1 might significantly contribute to the impairment detected by complex cognitive tests such as the Stroop (78). Recent data have highlighted similar concerns in studies of cognitive aging, noting that manual dexterity significantly contributes to performance in traditional paper-and-pencil assessments of cognitive processing speed in older adults (79). Interference from peripheral weakness would undermine use of such tools as CNS outcome measures in DM1 drug trials, since a therapy that successfully improves peripheral weakness or myotonia, and so increases reading aloud speed or manual dexterity, could erroneously give the impression of having impacted cognition. Our data demonstrate significant correlations of performance in key components of the Stroop, Trail Making Tests, and Block Design subtest with GMV after accounting for age and sex, suggesting structural brain changes are major modifiers of performance. However, inclusion of DM1-ActivC© score in a multivariate model supports the hypothesis that muscle impairment accounts for some of the residual variation in performance in the Stroop Color and Word tasks, as well as all elements of the Trail Making Tests. Perhaps unsurprisingly, the effects of muscle were most pronounced in the number scanning and motor components of the Trail Making Tests.

The Block Design test is also a common cognitive assessment, used primarily to assess visuospatial skills (80). Standard scoring systems are again heavily weighted to reward rapid completion of the designs, hence we hypothesized that distal muscle weakness in DM1 might account for a major portion of the deficit detected compared with controls. In this test however, we observed that the large effect size persisted despite elimination of weighting for speed in the non-adjusted score. Further, scores showed some positive correlation with the visuospatial and executive subscores of ECAS. These findings suggest the Block Design test is indeed sensitive for impairment of visuospatial cognition in DM1 patients. Although the non-adjusted score improved correlations with ePAL and GMV compared with the standard score, this should be interpreted with caution since this value could not be age-adjusted due to a lack of normative data.

Overall, our neuropsychology data suggest that there would be value in further work to determine the nature of the basic speed limitation detected in DM1 by the Stroop and Trail Making Tests, and in particular to distinguish whether this is related to peripheral muscle impairment or other central factors. Furthermore, development and validation of assessments that are not excessively influenced by manual dexterity or dysarthria, perhaps utilizing assistive technology, would also be a useful step toward clinical trial readiness.

In self-reported symptom questionnaires, we observed strong mutual correlations between symptoms of fatigue, pain and low mood, consistent with the model of inter-relatedness between mental wellbeing and physical symptoms in DM1 that has driven recent research into cognitive behavioral therapy-based interventions (81). Patients with lower mood reported more somatic symptoms in general, particularly relating to cognition and social performance. This self-reported impairment was not generally reflected in poorer performance in neuropsychological assessments and, similar to the observations of previous authors (35, 82), we found a trend toward greater reporting of depression and central symptoms in those with milder white matter change on MRI. It is unclear whether this trend reflects increasing acceptance of symptoms over time, or reduced awareness as has been previously described (51). In contrast, executive symptoms rated by a relative or carer showed a positive relationship with the severity of MRI changes. These data therefore suggest that self-reported symptoms alone are not an effective means to quantify the severity of the primary disease process in brain in DM1 study cohorts, and highlight a possible role for proxy measures as part of a global CNS assessment.

Since study visits for future clinical trials are likely to include additional effort-intensive measures of muscle symptoms as well as a CNS assessment protocol, it remains desirable to minimize redundant or duplicate outcome measures. It is therefore useful to note that, consistent with previous data (83), subscores within the MDHI correlated well with other measures of similar themes. This supports the MDHI as a good stand-alone measure of self-reported symptoms. This is with the caveat that, as outlined above, self-reported symptom scales may be influenced by mood or insight issues, and hence should be supported by objective measures of the relevant disease process where possible in the context of a clinical trial.

Correlations of CTG repeat length with neuropsychology assessments were comparatively poor. Several factors likely contribute to this observation, including relatively small cohort size, which may be subject to selection bias, and selection for adult onset DM1 only (excluding severe phenotypes and thus large repeat sizes). Because the cognitive phenotype in DM1 is generally one of mild impairment within the general population range, (in contrast to muscle weakness, which may be affected well outwith the range of normal variation), it may be that the effect of CTG repeat length on the multifactorial trait of cognition is too subtle to detect within the sample tested. A more marked effect of repeat length on muscle strength compared with central phenotypes is supported by the observation that the strongest genetic correlation observed in our cohort was with the MDHI mobility score.

With regard to genotype-phenotype correlations, it is also noteworthy that the three individuals identified with variant repeats reported particularly mild muscle impairment in DM1-ActivC ©. This adds to growing evidence that individuals with DM1 due to variant repeats may be statistical outliers in terms of disease severity (54), and so reinforces a role for robust genotyping, including screening for variant repeats, in DM1 clinical trials.

Although this study was not intended primarily to evaluate MRI biomarkers, it was interesting to note an apparent sex effect on GMV in this cohort. Inclusion of sex in a multivariate model improved both correlations of GMV with age, and with performance in several neuropsychology assessments, although stringent correction for multiple comparisons meant some could not be considered significant. To our knowledge, sex-specific differences in gray matter atrophy have not specifically been explored in DM1, but in the general population, a marginally greater rate of gray matter atrophy in males is observed (84). Therefore, given that several features of DM1 show a sex bias in penetrance (85), it is plausible that a sex effect on gray matter atrophy might exist in DM1. This finding highlights sex as an important cofactor to consider in future studies aiming to identify imaging biomarkers. Unlike global GMV, VWML did not correlate well with cognitive impairment measured by neuropsychology assessments, nor with CTG repeat length in this study. Given that white matter lesions in the general population may be influenced by vascular risk factors (86), it is probable that additional environmental and/or genetic factors also influence the severity of VWMLs in DM1, which may limit their potential for use as a disease-specific biomarker. Further studies in larger cohorts, using a variety of imaging modalities and regional structural measures as well as a longitudinal design are warranted to identify clinically meaningful imaging markers in DM1.

## Conclusions

This case control study applied a range of CNS measures with potential for use in clinical trials to 45 adults with DM1. Our data highlight muscle impairment and possible deficits in simple information processing as potential confounders of performance in complex neuropsychology assessments, particularly Trail Making and Stroop tests. We demonstrated that low mood is associated with greater self-reporting of central symptoms in general, and that significant depression appears to be more common in those with milder CNS involvement. An apparent sex effect was observed in volumetric analysis of global gray matter, which shows promise as a potential outcome measure, although further longitudinal studies in a larger population using a range of MRI modalities are indicated to identify and validate imaging biomarkers.

## Author contributions

MH, JMcL, CL, RJ, JE, DM, and MF contributed to the conception and design of the study. SC and JMcG generated and curated genetic data. BB contributed to identification of participants and interpretation of clinical data. JMcL and RJ undertook MRI planning and analysis. MH undertook clinical data collection, primary data analysis, and prepared the first manuscript draft, with guidance from SC, JE, MF, and DM. All authors contributed intellectually to subsequent redrafting of the manuscript.

### Conflict of interest statement

DM has been a paid scientific consultant and/or received honoraria from Biogen Idec, AMO Pharma, Charles River, and Vertex Pharmaceuticals. DM also has a research contract with AMO Pharma. JE is a co-author of the Behavioral Assessment of the Dysexecutive Syndrome test battery, and as such receives royalties from use of the Dysexecutive (DEX) Questionnaire. MH, JMcL, SC, BB, JMcG, RJ, CL, and MF report no relevant disclosures.

## Supplementary material

Supplementary File S1 Raw results data.

Supplementary File S2 Linear regression analyses and Benjamini-Hochberg correction.
